# Disrupted connectivity within visual, attentional and salience networks in the visual snow syndrome

**DOI:** 10.1002/hbm.25343

**Published:** 2021-01-15

**Authors:** Francesca Puledda, Owen O'Daly, Christoph Schankin, Dominic Ffytche, Steven CR Williams, Peter J Goadsby

**Affiliations:** ^1^ Headache Group, Department of Basic and Clinical Neuroscience King's College London London United Kingdom; ^2^ NIHR‐Wellcome Trust King's Clinical Research Facility SLaM NIHR Biomedical Research Centre, King's College Hospital London United Kingdom; ^3^ Centre for Neuroimaging Sciences, Department of Neuroimaging King's College London London United Kingdom; ^4^ Department of Neurology Inselspital, Bern University Hospital, University of Bern Bern Switzerland; ^5^ Department of Old Age Psychiatry Institute of Psychiatry, Psychology & Neuroscience, King's College London London United Kingdom

**Keywords:** functional connectivity, magnetic resonance imaging, migraine, photophobia, prolonged visual disturbance, salience network, visual motion network, visual snow syndrome

## Abstract

Here we investigate brain functional connectivity in patients with visual snow syndrome (VSS). Our main objective was to understand more about the underlying pathophysiology of this neurological syndrome. Twenty‐four patients with VSS and an equal number of gender and age‐matched healthy volunteers attended MRI sessions in which whole‐brain maps of functional connectivity were acquired under two conditions: at rest while watching a blank screen and during a visual paradigm consisting of a visual‐snow like stimulus. Eight unilateral seed regions were selected a priori based on previous observations and hypotheses; four seeds were placed in key anatomical areas of the visual pathways and the remaining were derived from a pre‐existing functional analysis. The between‐group analysis showed that patients with VSS had hyper and hypoconnectivity between key visual areas and the rest of the brain, both in the resting state and during a visual stimulation, compared with controls. We found altered connectivity internally within the visual network; between the thalamus/basal ganglia and the lingual gyrus; between the visual motion network and both the default mode and attentional networks. Further, patients with VSS presented decreased connectivity during external sensory input within the salience network, and between V5 and precuneus. Our results suggest that VSS is characterised by a widespread disturbance in the functional connectivity of several brain systems. This dysfunction involves the pre‐cortical and cortical visual pathways, the visual motion network, the attentional networks and finally the salience network; further, it represents evidence of ongoing alterations both at rest and during visual stimulus processing.

## Graphical abstract

This study investigates the functional connectivity of visual snow syndrome, compared to that of healthy controls. The results suggest that VSS is characterised by a widespread dysfunction in the functional connectivity of several brain systems, mostly the pre‐cortical and cortical visual pathways, the visual motion network, the attentional networks and the salience network. The investigation shows evidence of ongoing alterations both at rest and during visual stimulus processing in this poorly‐known and disabling neurological condition.

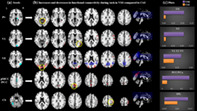

## 1 INTRODUCTION

Visual snow is a neurological condition consisting of a constant positive visual disturbance described as tiny flickering dots covering the entire field of vision. Patients can often experience other visual symptoms in addition to the static phenomenon. These are palinopsia, photophobia, entoptic phenomena and nyctalopia, which in different combinations constitute the visual snow syndrome (VSS) (Schankin, Maniyar, Digre, & Goadsby, 2014). Visual snow represents a spectrum type disorder that at its worse manifests with most, if not all, additional symptoms, as well as with distressing comorbidities such as migraine and tinnitus (Puledda, Schankin, & Goadsby, 2020). VSS can be misdiagnosed as hallucinogenic persisting perceptual disorder, which sometimes presents similar clinical features (Halpern, Lerner, & Passie, 2018); the two, however, are clearly separate conditions (Puledda, Schankin, Digre, & Goadsby, 2018).

The pathophysiology underlying visual snow is largely unknown. Several hypotheses have been proposed, including a dysrhythmia of the thalamo‐cortical pathways (Lauschke, Plant, & Fraser, 2016), increased cortical excitability in higher order visual processing areas (Eren, Rauschel, Ruscheweyh, Straube, & Schankin, 2018; Luna, Lai, & Harris, 2018; McKendrick et al., 2017; Yildiz, Turkyilmaz, & Unal‐Cevik, 2019), heightened saliency of normally ignored subcortical activity, or a combination of all these mechanisms (Puledda, Ffytche, O'Daly, & Goadsby, 2019).

To date few neuroimaging studies have examined the central neurobiology of visual snow. One study integrating [^18^F]‐FDG PET to investigate brain metabolism of VSS patients and voxel‐based morphometry with MRI compared with controls, demonstrated hypermetabolism and increased cortical volume in the extrastriate visual cortex at the junction of the right lingual and fusiform gyrus (Schankin et al., 2020). In another study using ^1^H‐MRS over the right lingual cortex, lactate concentrations were increased in patients with VSS compared with controls suggesting a localised disturbance of anaerobic metabolism (Puledda et al., 2020).

In the present study, we investigated the functional connectivity of patients with VSS using resting state functional magnetic resonance imaging (fMRI) with multi‐echo planar imaging, an emerging approach which allows us to more readily distinguish true BOLD signal fluctuation from artefact signal with higher fidelity (Kundu et al., 2017).

We defined eight seeds based on previous hypotheses and following anatomical areas of interest. Four seeds were placed unilaterally in key regions within the pre‐cortical and cortical visual pathways. These were: the right pulvinar, an area of the thalamic matrix that plays a significant role in cognition and attentive stimulus processing (Lakatos, O'Connell, & Barczak, 2016), as well as photophobia (Maleki, Becerra, Upadhyay, Burstein, & Borsook, 2012) one of the chief symptom of VSS (Eren, Ruscheweyh, Straube, & Schankin, 2019); the right primary visual area V1, the earliest cortical area for visual stimulus processing; the right motion area V5 which specifically detects motion stimuli (Zeki et al., 1991) and is therefore relevant in a condition characterised by the constant perception of small moving dots; and finally the right lingual gyrus, which was the first area to be implicated directly in VSS pathophysiology (Schankin et al., 2014). The reason for choosing the right hemisphere over the left was specifically due to the previous finding of right lingual gyrus involvement in VSS pathophysiology. The remaining four regions of interest were derived from a pre‐existing functional analysis using pseudo‐continuous arterial spin labelling and performed on the same patient cohort (Puledda, Zelaya, Schankin, & Goadsby, 2018). These were localised at the levels of lobule VI in the left cerebellum, the posterior midcingulate and posterior cingulate cortices, the left parietal lobule and precuneus, and the right anterior insula. As they form part of larger brain networks, such as the default mode (DMN) and dorsal attentional networks (DAN), these structures were also of interest for their involvement in higher‐order sensory processing and complex cognitive tasks.

Finally, all subjects were investigated in both a resting state and a stimulated condition, through the use of a visual task that simulated the visual snow percept. As VSS is characterised by an abnormal perceptual experience, it was considered relevant to be able to differentiate if changes in brain function could be due to altered visual processing, rather than to the ongoing process of a constant stimulation caused by the ‘visual snow’ effect itself.

## 2 MATERIALS AND METHODS

### 2.1 Subject recruitment and study protocol

An equal number of patients with VSS diagnosed according to the current criteria (Schankin, Maniyar, Digre, & Goadsby, 2014) and healthy volunteers selected to match the age (± 5 years) and gender of the patient group were recruited.

We recruited VSS patients by email, re‐approaching subjects who had previously contacted our study team asking to participate in research studies. Healthy volunteers were recruited through internal advertisement at King's College London.

Participants had to be between 20 and 60 years old, with no contraindications to undergo an MRI, no serious previous medical conditions, no history of any recreational drug intake in the past, consumption of no more than six cups of coffee per day, no recurrent medication intake with an action on the central nervous system, no psychological diseases that would require medication or that could affect neural pathways.

All participants gave their informed consent. The study was approved by the London ‐ City & East Research Ethics Committee (Reference number: 16/LO/0964).

Participants were first contacted by telephone in order to assess their eligibility. Following this, they were invited to either one or two visits to our research facility, depending on whether they were in the control or patient group, respectively. During the first visit, patients underwent a full medical history taking, as well as a general examination and a neurological examination, blood pressure and heart rate monitoring. During the second visit the scanning, lasting ~70 min, took place. Controls came only for the scanning visit. All participants were scanned at the same time of day (between 9 and 12 a.m.) (Hodkinson et al., 2014). Subjects were instructed to consume a light breakfast and to avoid caffeine prior to the visit. Participants were asked to refrain from the use of any type of medication for 24 hr prior to scanning. To ensure patients were not scanned during the acute migraine phase, they were instructed to inform the investigators if a migraine attack was experienced in the 48 hr prior and following the imaging visit. This was further verified during the visit itself by the investigator and by email follow‐up.

### 2.2 Magnetic resonance imaging

Scanning was performed on a 3 T General Electric MR750 MRI scanner at the NIHR‐Wellcome Trust King's Clinical Research Facility, King's College Hospital, London using a 12‐channel head coil. The scanning protocol was the same for both groups and was conducted over a single session.

For fMRI, participants underwent two acquisitions, a baseline and a stimulation scan. In the baseline scan, all participants were lying still with their eyes open while looking at a blank screen, which they viewed through a mirror system. For the second acquisition, all participants were subjected to a visual task that mimicked the static of visual snow, shown continuously through the same screen. The development of the visual task has been explained in detail elsewhere (Puledda, Ffytche, et al., 2020).

Total scanning time for each fMRI acquisition (at rest and during visual stimulation) was 10 min. During this time, multi‐echo EPI images sensitive to BOLD contrast were acquired to measure hemodynamic responses with the following characteristics: TR = 2,500 ms; echo times = 12, 28, 44, 60 ms; flip angle = 80°; FOV = 240 × 240 mm; matrix = 64 × 64; slice thickness = 3 mm; 32 axial sections collected with sequential (top down) acquisition and 1 mm interslice gap; in‐plane resolution = 3.75 mm.

A high‐resolution structural scan was acquired for co‐registration of the ME‐EPI data by means of a three‐dimensional 3D T1‐weighted IR‐SPGR image. The parameters of this scan were: TR = 7.312 ms; TE = 3.016 ms; TI = 400 ms; flip angle = 11°; FOV = 270 × 270 mm; matrix = 256 × 256; slice thickness = 1.2 mm; 196 slice partitions, ASSET factor = 1.75; in‐plane resolution = 1 mm (Jack Jr. et al., 2010).

### 2.3 Image processing

Unless otherwise stated, all fMRI data were processed and analysed using Statistical Parametric Mapping software suite, version 12 (SPM 12; www.fil.ion.ucl.ac.uk/spm/) in MATLAB R2017a (https://uk.mathworks.com/). After resetting of the origins for both T1‐weighted and ME‐EP images, the 60 ms echo was discarded due to low signal‐to‐noise ratio. The remaining three echoes were taken forward to preprocessing as described below. The ME‐EPI echoes were separated into distinct time series (corresponding to the three remaining individual echoes), which were then de‐spiked using 3D Despike in the Analysis of Functional NeuroImages (AFNI) framework (https://afni.nimh.nih.gov), and slice time corrected. Parameters for motion correction were estimated for the first echo, and subsequently applied to the other echoes; all ME‐EP images were then co‐registered to the T1 scan. All echoes were spatially normalised to the study‐specific template, and from there to Montreal Neurological Institute (MNI) space. Finally, the images from all echoes were z‐concatenated for further processing, that is, the space‐bytime matrices from each echo were appended to one another in the z‐direction to form a single matrix using the *3dZcat* function in AFNI. TEDANA, a python script that forms part of the Multi Echo Independent Component Analysis (MEICA) package (https://afni.nimh.nih.gov/pub/dist/src/pkundu/meica.py) (Kundu et al., 2017; Kundu, Inati, Evans, Luh, & Bandettini, 2012) was called to perform TE dependent ICA‐based denoising and T2* weighted averaging (optimal combination) of echoes. Motion correction, white matter signal and cerebrospinal fluid signal were regressed out from the denoised, optimally combined images; subsequently the residual time series were band‐pass‐filtered with AFNI (frequency range 0.08–0.01 Hz).

### 2.4 Imaging analysis

**FIGURE 1:**
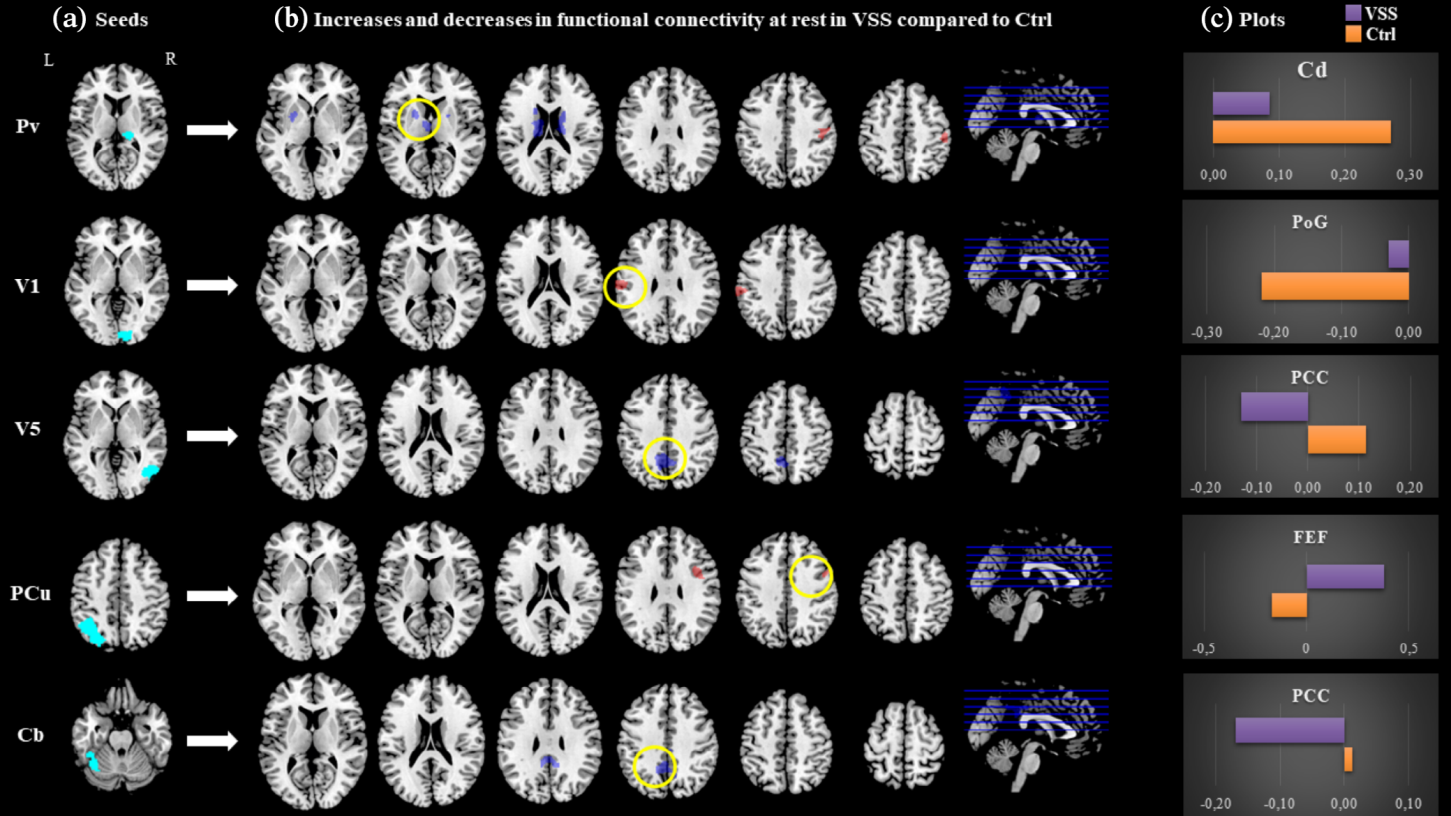
Main resting state connectivity differences between visual snow patients and healthy controls. (a) Location of selected seed regions (in cyan). (b) Between‐group functional connectivity maps from each seed region to the whole brain in VSS patients compared with controls. Blue refers to reduced coupling compared with Ctrls, red refers to increased coupling. Maps are thresholded at *P* < .001 and cluster corrected to *P* < .05. For T and *k* values refer to Table 2. (c) Plots for clusters with highest *T* value (circled in yellow), with respective bar charts of beta values for the two groups (VSS in purple, Ctrl in orange). Pv, pulvinar; V1, right primary visual area; V5, right V5 area; PCu, precuneus; Cb, left cerebellum lobule VI; Cd, caudate nucleus; PoG, postcentral gyrus; PCC, posterior cingulate cortex; FEF, frontal eye field

**FIGURE 2:**
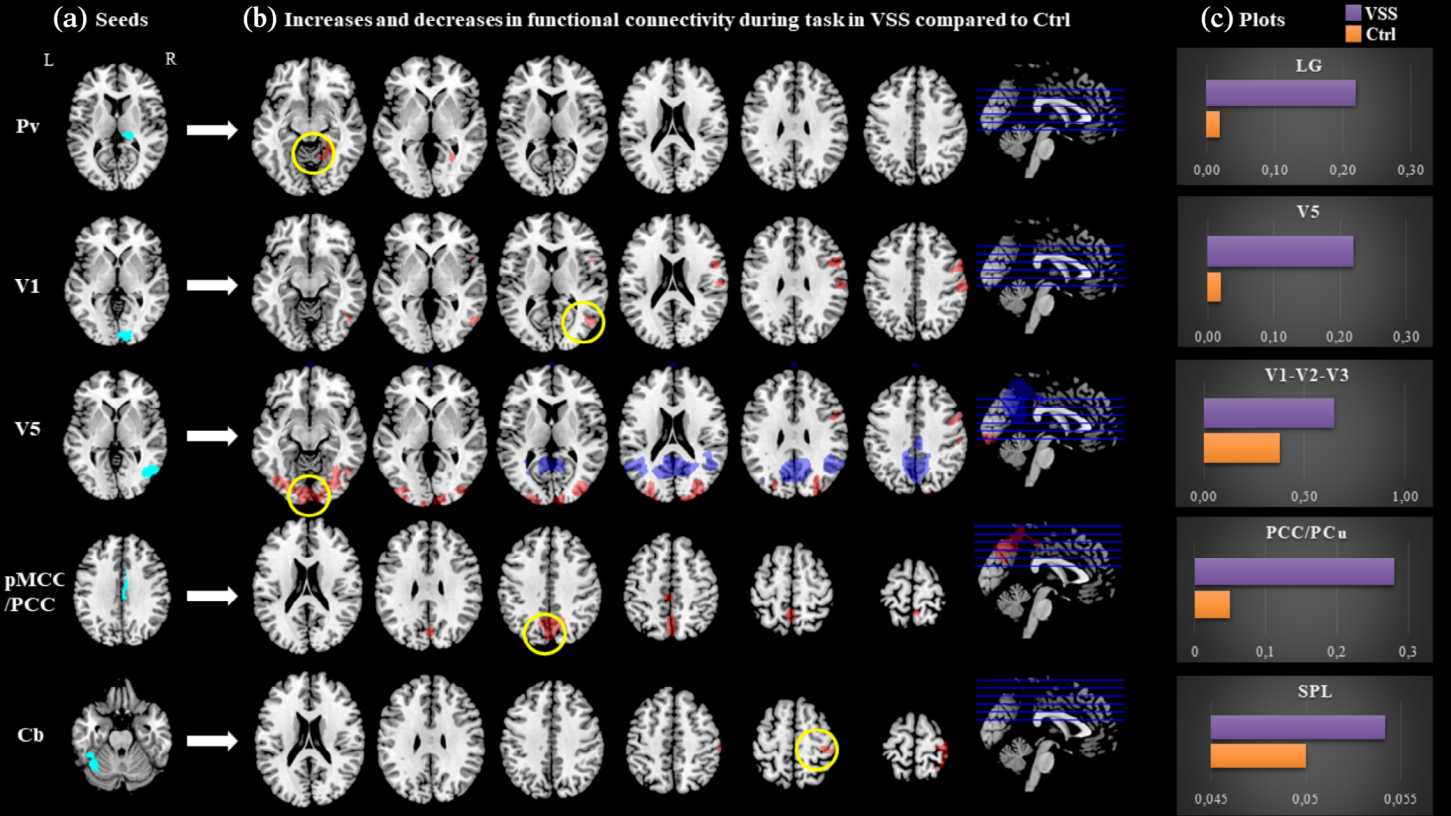
Main task‐based connectivity differences between VSS patients and healthy controls. (a) Location of selected seed regions (in cyan). (b) Between‐group functional connectivity maps from each seed region to the whole brain in VSS patients compared with controls. Blue refers to reduced coupling compared with Ctrls, red refers to increased coupling. Maps are thresholded at *P* < .001 and cluster corrected to *P* < .05. For T and *k* values refer to Table 2. (c) Plots for clusters with highest T value (circled in yellow), with respective bar charts of beta values for the two groups (VSS in purple, Ctrl in orange). Pv, pulvinar; V1, right primary visual area; V5, right V5 area; pMCC/PCC, posterior midcingulate cortex/posterior cingulate cortex; Cb, left cerebellum lobule VI; LG, lingual gyrus; SPL, superior parietal lobule

As detailed in the introduction, we defined eight unilateral seeds based on previous hypotheses and following anatomical areas of interest in the visual network: right pulvinar (Pv); right primary visual area V1 (V1); right motion area V5 (V5); right lingual gyrus (LG); left cerebellum lobule VI (Cb); posterior midcingulate cortex/posterior cingulate cortex (pMCC/PCC); left precuneus (PCu) and right insula (IN) (Figures 1a and 2a). The pMCC/PCC, PCu, Cb and IN regions‐of‐interest (ROIs) were created as binary masks from positive clusters derived from the results of a preliminary pseudo‐continuous arterial spin labelling analysis. Anatomical ROIs for the remaining areas were created using the ‘wfu_pickupatlas Anatomical Library’ (https://www.nitrc.org/projects/wfu_pickatlas/), as implemented in the SPM toolbox, except for the V5 ROI which was created from the ‘Juelich Histological Atlas’ within FSLeyes. Detailed images for the eight seeds, as well as coordinates and sizes, can be found in the supplementary material.

For each seed region the mean time series was extracted from the band‐pass filtered data and a voxel‐wise Pearson's correlation was then calculated. The connectivity maps were taken forward to the subsequent group level analysis. The resting and visual task connectivity data were analysed using a voxel‐wise general linear model in SPM 12. A second‐level whole brain voxel‐wise flexible‐factorial design using two‐way ANOVA allowed analysis of changes in connectivity related to group and stimulus effect. We also performed post hoc analyses with migraine as a covariate, to account for the high comorbidity with this disorder in our patient population.

For all voxel‐wise analyses, significance was defined with an initial cluster‐forming voxel threshold of *P* < .001 and family‐wise error (FWE) correction, on the basis of cluster extent, to *P* < .05, using the Gaussian random field theory. Having analysed multiple ROIs and two different conditions (at rest and during visual task) for each region, adjusted *P* thresholds corrected for FDR of 5% (following the Benjamini‐Hochberg's procedure) were calculated in order to account for multiple comparisons correction.

All brain locations are reported as x, y and z coordinates in Montreal Neurologic Institute (MNI) space. A neuroanatomy atlas (Mai, Paxinos, & Voss, 2008), as well as the Harvard‐Oxford cortical and subcortical structural atlases from the FSL software (FSL 5; https://fsl.fmrib.ox.ac.uk/fsl/fslwiki), were used to identify the correct anatomical locations of clusters of statistically significant changes within MNI space.

## 3 RESULTS

### 3.1 Demographic and clinical data

A total of *n* = 24 patients with visual snow and *n* = 24 healthy controls (Ctrl) took part in the study. Mean age for the VSS group and Ctrl group was 28 ± 6 and 28 ± 5 years, respectively (*P* = .8). The VSS group had an equal number of female and male participants (12 for each group) and the Ctrl group had 14 females and 10 males; the difference between the two groups was not significant (*P* = .6). Handedness was also balanced between the two cohorts (right:left ratio VSS group 21:3; control group 23:1; *P* = .3). Clinical and demographic details of the VSS population can be found in Table 1. No change in the intensity or pattern of visual snow symptoms was recorded for any patient in‐between visits.

**TABLE 1:** Main features of VSS patient group, showing gender, age at time of the study, age at visual snow onset, type of static, additional visual symptoms, main comorbidities and concomitant medication taken at the time of the study

Gender	Age (years)	Onset of VS (age)	Static type	Additional symptoms								Comorbidities			Concomitant medication
				A	T	BFEP	FL	SL	FLA	NY	PH	TIN	MIG	AUR	
F	33	#	BW,C,F,T	+	+	+	+	+	+	+	+	+	+	+	Multivitamins
M	28	10	C	+				+				+			
M	29	26	BW	+		+	+	+	+		+	+	+		Levothyroxine, paracetamol on occasion
M	25	19	BW,F,T	+	+	+	+	+	+	+	+	+	+		
F	20	#	BW,T	+		+	+	+	+		+	+	+		Fexofenadine
M	31	9	BW,F	+		+	+		+	+		+			Pimecrolimus topical, betamethasone topical
F	34	#	BW,C,F	+		+		+	+	+			+	+	
F	23	#	BW,C	+	+		+	+	+	+			+		Oral contraceptive pill
F	21	#	BW,F,T	+	+	+	+	+	+	+	+	+	+	+	Paracetamol on occasion
M	27	21	BW	+	+	+	+	+	+		+				
F	26	26	BW	+	+	+	+	+	+	+	+	+			Multivitamins, ibuprofen on occasion
F	43	43	BW,F,T	+		+	+	+		+		+			
F	34	12	BW		+	+	+	+		+	+	+	+	+	
F	22	#	T	+		+	+	+	+			+	+		Multivitamins, Paracetamol, Nexplanon
F	34	31	BW,T	+	+	+	+	+	+	+	+	+	+	+	
M	22	15	F,T			+	+	+		+					
F	25	#	T	+	+	+	+	+			+	+			Salbutamol inhaler, multivitamins
F	26	25	BW,C,F,T	+	+	+		+	+	+	+	+	+	+	Paracetamol on occasion
M	22	17	F	+		+	+			+	+	+	+		Magnesium
M	31	24	BW	+	+	+	+	+	+		+		+	+	Paracetamol on occasion
M	35	33	BW	+	+	+	+	+		+	+	+	+		Levothyroxine, CQ10
M	19	#	BW				+				+	+			
M	29	#	F,T		+	+	+	+				+			
M	30	#	BW,F	+	+	+	+	+	+	+		+	+		Fluticasone nasal spray

*Note*: = Symptoms present for as long as patient could recall; + = present.

Abbreviations: A, afterimages; AUR, visual aura; BFEP, blue‐field entoptic phenomena; BW, black and white static; C, coloured static; F, flashing static; FL, floaters; FLA, flashes; MIG, migraine; NY, nyctalopia; PHO, photophobia; SL, self‐light of the eye; T, trailing; T, transparent static; TIN, tinnitus.

### 3.2 Functional connectivity analysis: Group effects

To test our hypothesis that individuals with VSS would show altered functional connectivity of the visual network compared with Ctrls, following Fisher's r‐to‐Z transformation of connectivity r‐maps, we compared whole‐brain connectivity for each visual network seed (Pv, LG, V1, V5) between groups, both during the resting state and in the presence of the visual stimulus. This analysis revealed hyper and hypoconnectivity in VSS compared with Ctrls between key visual areas and the rest of the brain, as outlined below; changes were detected in either the resting or activated states, or both. No difference in functional connectivity between groups was found in the whole‐brain analysis for the LG seed. We also tested the whole‐brain connectivity of the pMCC/PCC, precuneus and lobule VI of the left cerebellum. These structures are respectively part of the default mode network (PCC/PCu) and the dorsal attentional (Cb) networks, and are implicated in important sensory and cognitive tasks.

A summary of significant areas of increased and decreased connectivity for the main effect of group, both at rest (Figure 1) and during the task condition (Figure 2), between these defined ROIs and the rest of the brain is described in Table 2 and as follows.

**TABLE 2:** Areas of increased and decreased connectivity for main effect of group (VSS patients vs. Ctrl), at rest and during the visual task, from selected ROIs to the rest of the brain

ROI	Condition	Contrast	Brain regions and Brodmann areas	Mean beta values		*P* (FWE)	*P* (FDR)	k	T	Peak coordinates		
				VSS	Ctrl					x	y	z
Pv	Rest	VSS > Ctrl	R SMG and postcentral gyrus (BA 1,3)	−0.01	−0.18	.05	.05	382	4.32	50	−20	46
		VSS < Ctrl	Bilateral caudate nuclei^a^	0.09	0.27	≤ .001	.01	967	4.68	−12	−6	16
	Task	VSS > Ctrl	R LG (BA 19)^a^	0.22	0.02	.04	.04	410	4.27	26	−54	−2
V1	Rest	VSS > Ctrl	L SMG and postcentral gyrus (BA 1,3)^b^	−0.03	−0.22	.05	.05	346	4.54	−64	−20	32
	Task	VSS > Ctrl	R IOG (area V5, BA 18,19)^b^	0.24	0.01	.05	.05	332	5.02	52	−64	6
			R SMG and postcentral gyrus (BA 1,3)^b^	0.00	−0.19	.007	.03	572	4.68	58	−24	40
			R precentral gyrus (BA 8, 6, FEF)^a^	0.08	−0.13	.003	.02	696	4.53	52	10	28
V5	Rest	VSS < Ctrl	PCC, L/R medial precuneus (BA 7)^b^	−0.13	0.11	.01	.04	554	4.46	−6	−52	42
	Task	VSS > Ctrl	Bilateral MOG, SOG, IOG, FG, (BA 17, 18, 19, V1‐V3), SMG, cuneus^b^	0.65	0.37	≤ .001	.01	5,598	5.71	38	−84	10
			R SPL/intraparietal sulcus (BA 7, BA5)^b^	0.53	0.27	≤ .001	.01	936	5.0	26	−60	60
			R precentral gyrus (BA 8, 6, FEF)^b^	0.41	0.16	.02	.04	420	4.25	48	2	38
		VSS < Ctrl	PCC, bilateral medial precuneus^b^	−0.25	0.00	≤ .001	.01	6,838	6.16	−4	−52	52
			R TPJ and AG (BA 39, BA 40)^b^	−0.17	0.05	≤ .001	.01	928	5.12	44	−56	28
PCu	Rest	VSS > Ctrl	R precentral gyrus (BA 8, 6, FEF)	0.38	−0.17	.004	.03	337	4.37	48	4	36
PCC/pMCC	Task	VSS > Ctrl	PCC, bilateral medial precuneus^b^	0.28	0.05	≤ .001	.01	1,535	4.12	−4	−50	58
Cb	Rest	VSS < Ctrl	PCC, bilateral medial precuneus	−0.17	0.01	.007	0.03	605	4.44	−10	−50	38
	Task	VSS > Ctrl	R SPL/lateral precuneus, postcentral gyrus (BA 2, 1,3)	0.05	−0.16	.004	.03	685	4.24	36	−40	72

*Note*: Clusters are shown in coordinate MNI space with relative *T* scores and *k* values. An initial voxel threshold of *P* < .001 and cluster correction to *P* < .05 was applied. Adjusted *P* thresholds following FDR correction for multiple testing are also shown; all clusters survived correction. Mean beta values for the clusters are shown for VSS and Ctrls. a and b indicate clusters that survived significance in a post hoc analysis run with migraine presence as a covariate.

Abbreviations: AG, angular gyrus; BA, Brodmann area; Cb, left cerebellum lobule VI; FEF, frontal eye field; FG, fusiform gyrus; IOG, inferior occipital gyrus; L, left; LG, lingual gyrus; MOG, middle occipital gyrus; PCC, posterior cingulate cortex; PCC, posterior cingulate cortex; PCu, left precuneus; pMCC, posterior mid‐cingulate cortex; Pv, right pulvinar; R, right; SMA, supplementary motor area; SMG, supramarginal gyrus; SOG, superior occipital gyrus; SPL, superior parietal lobule; TPJ, temporoparietal junction; V1, right primary visual area; V5, right V5 area.

^a^ Cluster present in post hoc analysis covarying for migraine presence, with reduced cluster forming threshold of *P* = .005.

^b^ Cluster present in post hoc analysis covarying for migraine presence.

### 3.3 Pulvinar

Compared with the healthy controls, VSS patients in the rest condition showed a greater connectivity between the right pulvinar and the right postcentral gyrus (PoG) and supramarginal gyrus (SMG) (T = 4.32; k = 382; *P* = .05; x = 50 y = −20 z = 46). When determining the effects of interest and examining the mean beta values of each group, we ascertained that VSS patients had a close to null coupling between these two areas, where controls showed anti‐correlation (i.e., negative connectivity values, Table 2). We also found a significantly reduced connectivity between the Pv and the bilateral caudate nuclei (T = 4.68; k = 967; *P* ≤ .001; x = −12 y = −6 z = 16; Figure 1) in VSS patients with respect to Ctrls.

When exposed to the task condition, patients with VSS showed a significantly positive coupling between the Pv and the right lingual gyrus (T = 4.27; k = 410; *P* = .04; x = 26 y = −54 z = −2; Figure 2), which was conversely close to null in Ctrls.

### 3.4 V1

In the VSS group, compared with Ctrls, we found evidence of greater connectivity at rest between the right primary visual cortex and the left SMG and postcentral gyrus (T = 4.54; k = 346; *P* = .05; x = −64 y = −20 z = 32; Figure 1). When lowering the statistical threshold for exploratory purposes, this area included the frontal eye fields (FEFs) and was present on the contralateral hemisphere as well.

During the task condition, patients with VSS exhibited significantly greater coupling than controls in the right V5 area (T = 5.02; k = 332; *P* = .05; x = 52 y = −64 z = 6; Figure 2) and positive or null connectivity rather than anti‐correlation in the postcentral and precentral gyri, SMG, premotor cortex, supplementary motor cortex (SMA) and FEFs of the same hemisphere (Table 2).

### 3.5 V5

At rest, patients with VSS exhibited significantly reduced connectivity from the right V5 area to the posterior cingulate cortex (T = 4.46; k = 554; *P* = .01; x = −6 y = −52 z = 42; Figure 1). Examining the effects of interest showed that, while the connection between these two areas was positive in controls, it was negative in VSS patients.

In the visually activated state, we found greater connectivity from V5 to several bilateral occipital, parietal and frontal areas, specifically the right cuneus and precuneus, Brodmann visual areas 17, 18 and 19, the FEF, SMG, premotor cortex, SMA, superior parietal lobule (SPL) and intraparietal sulcus (IPS) (Figure 2, Table 2). These areas also showed positive coupling in controls, however, the connection was significantly stronger in VSS patients.

During the task, anti‐correlation with the PCC was confirmed as in the resting state, and was found as well between V5 and the right temporo‐parietal junction (TPJ) (T = 5.12; k = 928; *P* ≤ .001; x = 44 y = −56 z = 28).

### 3.6 PCu

At rest, we found a significantly positive connectivity between the left precuneus and the right precentral gyrus/frontal eye fields (T = 4.37; k = 337; *P* = .004; x = 48 y = 4 z = 36), as opposed to the negative connectivity between these areas found in Ctrls (Figure 1).

### 3.7 PCC

There was a significantly stronger connectivity in the task state from the pMCC/PCC to the bilateral medial precuneus and PCC itself, in VSS patients with respect to Ctrls (Figure 2).

### 3.8 Cerebellum

From the cerebellar seed, VSS patients in the rest condition showed anti‐correlation to the PCC and medial precuneus (Figure 1), largely overlapping the area found to have reduced connectivity with the V5 region.

Conversely, during the task, there was greater coupling to the right SPL, lateral precuneus and PoG (Figure 2).

Post hoc analyses covarying for migraine presence revealed that most of the aforementioned clusters survived significance, albeit occasionally requiring a lower cluster‐forming threshold of *P* = .005, except for the ones from the cerebellar seed (Table 2).

A further post hoc analysis to investigate contralateral connectivity (not accounting for migraine presence) was performed on the anatomical regions of interest (Pv, V1, V5 and LG); details can be found in Table 3 and supplementary material. In summary, this analysis showed largely symmetric VSS‐driven differences for the main results. Specifically, there was symmetry to the main analysis in the decreased connectivity found between Pv and the caudate nucleus during the visual task, in the increased connectivity found at rest between V1 and SMG/PoG, in the increased connectivity during the activated state between V5 and V1‐V2‐V3, and finally in the decreased connectivity between V5 and the PCC/bilateral precuneus. Notably, regions that did not show symmetry and rather remained in the ipsilateral hemisphere with respect to the main analysis involved the increased connectivity at rest between Pv and PoG, which remained localised to the right hemisphere even from the left Pv seed, as well as the anticorrelation between V5 and right TPJ.

**TABLE 3:** Post hoc analysis investigating contralateral connectivity from the main anatomical regions of interest (Pv, V1, V5 and LG) and comparison to main results

ROI	Condition	Contrast	Brain regions and Brodmann areas	*P*	k	T	Peak coordinates			Comments and comparison to main analysis
							x	y	z	
Pv	Rest	VSS > Ctrl	R SMG and postcentral gyrus (BA 1,3)	.05	360	4.30	52	−20	46	Same cluster as main analysis, remains ipsilateral
		VSS < Ctrl	Left caudate nucleus^a^	.02	1,148	4.71	−12	−6	16	Only left‐sided cluster survives
V1	Rest	VSS > Ctrl	R SMG and postcentral gyrus (BA 1,3)^a^ ^b^	.02	983	3.94	60	6	40	Same cluster as main analysis, contralateral hemisphere.
	Task	VSS > Ctrl	R SMG and postcentral gyrus (BA 1,3)	.02	443	4.43	54	−18	32	Same cluster as main analysis, remains ipsilateral
			R precentral gyrus (BA 8, 6, FEF)	.001	822	5.06	52	10	28	Same cluster as main analysis, remains ipsilateral
V5	Task	VSS > Ctrl	Left MOG, SOG, IOG, FG, (BA 17, 18, 19, V1–V3), SMG, cuneus^a^ ^b^	.006	1,241	4.3	38	−82	4	Same cluster as main analysis, lateralized to left hemisphere
			R SPL/intraparietal sulcus (BA 7, BA5)^b^	.04	800	3.99	26	−80	32	Same cluster as main analysis, remains ipsilateral
		VSS < Ctrl	PCC, bilateral medial precuneus^a^	≤ .001	1805	4.67	12	−60	36	Same mid‐line and bilateral clusters as in main analysis
			R TPJ and AG (BA 39, BA 40)	.05	463	4.47	44	−44	26	Same cluster as main analysis, remains ipsilateral

^a^ Clusters that showed symmetry to the main analysis.

^b^ Regions for which cluster forming threshold was lowered to *P* = .005.

### 3.9 Functional connectivity analysis: Interaction effects

**FIGURE 3:**
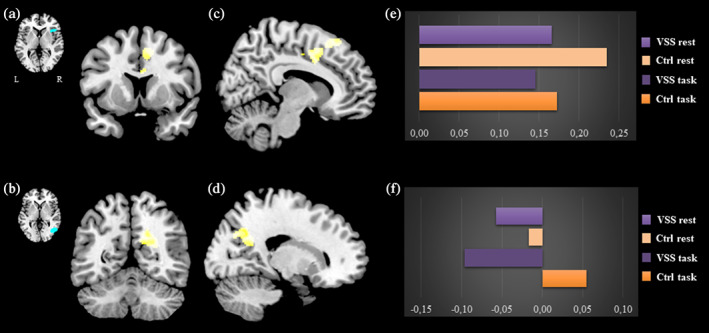
Interaction effects of functional connectivity between groups (VSS patients vs. Ctrls) and conditions (rest vs. task). The image shows a cluster of decreased task‐related connectivity between the right insula seed (a) and the anterior‐middle cingulate cortex (c) and between the V5 seed (b) and the right precuneus (d). On the right, plots for the beta values of the conditions (E and F, respectively) are shown. For the insula to ACC cluster (C): T = 4.83; *k* = 505; *P* = . 009; x = 2 y = 2 z = 30. For the V5 to precuneus cluster (D): T = 5.29; *k* = 440; *P* = .01; x = 24 y = −54 z = 24. Maps are thresholded at *P* < .001 and cluster corrected to *P* < .05

When analysing the interaction effect between group (i.e., VSS vs. Ctrls) and stimulus condition (i.e., rest vs. visual task), we found a decreased task‐related connectivity between the right anterior insula and the anterior‐middle cingulate cortex (ACC) (T = 4.83; k = 505; *P* = . 009; x = 2 y = 2 z = 30) and between V5 and the right precuneus (T = 5.29; k = 440; *P* = .01; x = 24 y = −54 z = 24), in VSS patients with respect to controls (Figure 3a–d).

Specifically, as can be seen in Figure 3e, there was a reduction in the insula to ACC connectivity between the rest and task condition in both groups, however, this was significantly stronger in Ctrls when compared with the VSS patients.

Connectivity from V5 to the right precuneus, on the other hand (Figure 3f), showed an opposite behaviour between rest and task in the two groups. Although both VSS and Ctrls showed anti‐correlation between the two areas at rest, during the task the VSS group showed a stimulus‐related strengthening of this anti‐correlation, whereas Ctrls switched to an increased connectivity during the stimulated state.

## 4 DISCUSSION

The data demonstrate a widespread disturbance in functional connectivity characterising the visual snow brain. First, several regions within the visual network show altered connectivity amongst themselves, as well as with numerous other important areas, such as the basal ganglia, FEFs and attentional networks. Secondly, key elements of the DMN and salience network also presented relevant disruptions of functional coupling. Thirdly, connectivity between certain brain regions exhibits an opposite response to an external visual task in VSS, with respect to healthy subjects. Taken together, these findings suggest that both during the resting state and in an activated brain condition, VSS is characterised by a reorganisation of the coupling within functional brain networks.

### 4.1 Pre‐cortical visual pathways

The finding that in VSS at rest the pulvinar, essential thalamic hub for cognition and visual stimulus processing (Lakatos et al., 2016; Robinson & Petersen, 1992) has reduced connectivity to the bilateral dorsal aspects of the caudate nuclei, is relevant. The tail of the caudate nucleus is part of the visual cortico‐striatal loop (VCSL), which has a role in visual learning, by selecting and reinforcing relevant peripheral stimuli for further processing and by conversely inhibiting irrelevant ‘error’ stimulations (Alexander, DeLong, & Strick, 1986; Seger, 2013), through a form of feedforward predictive coding (Friston & Kiebel, 2009; Rao & Ballard, 1999). A bottom‐up disruption of the circuitry, such as we found in VSS patients, could potentially allow for incorrect ascending noise‐like information to reach higher hierarchical levels, thus creating a mismatch between the default prediction of the world and a noise‐like perception that would normally be cancelled out by the brain.

Our results also showed that during the visual task, post‐thalamic visual pathways from the right pulvinar to the right lingual gyrus have strengthened connectivity in patients with VSS. This heightened connection could explain the symptom of photophobia, given that the both the pulvinar (Schwedt et al., 2013) and the LG (Denuelle et al., 2011) have shown a significant involvement in this symptom in previous studies on migraineurs. This result further provides an explanation of the VSS‐related increased metabolism in the right LG found with [^18^F]‐FDG PET (Schankin et al., 2020), and reinforced by lactate accumulation in that brain area (Puledda, Ffytche, et al., 2020). In this context, the specific involvement of the right lingual gyrus in VSS from previous studies would partially explain why our result was not confirmed in the contralateral hemisphere, although a full explanation for this lateralization cannot be drawn from this analysis alone. Finally, an increased connectivity of the pre‐cortical visual pathways could also be one of the underlying phenomena driving a wider cortical network dysfunction in VSS, ultimately causing reduced filtering of incoming visual information.

### 4.2 Striate visual cortex involvement

The primary visual cortex in VSS patients showed increased coupling with the homolateral FEFs, the SMG, the premotor cortex and the SMA, as opposed to what was found in healthy subjects. This heightened connectivity was observed primarily in the presence of the visual stimulus, however lowering the statistical threshold allowed to ascertain that it was a feature of the resting condition as well. The FEFs and SMG are implicated in visual attention and in the generation of active saccades (Schall, 2004) and antisaccades (Ettinger et al., 2007; Vernet, Quentin, Chanes, Mitsumasu, & Valero‐Cabré, 2014). An opposite connection between the visual cortex and these regions could represent an alteration of the physiological processes of relevant stimulus selection in VSS patients.

Further, VSS patients showed a powerful increase in the connectivity between V1 and V5, in the context of the visual stimulus. An increased effective connection between dorsal parietal areas and V1 could represent a gain of function within the visual motion network, which would also justify recent findings of increased GM volume in both V1 and V5 in patients with VSS (Puledda et al., 2020); it could also represent a strengthening of the dorsal visual stream, a system involved in the integration of vision and proprioception, essential in determining action‐oriented behaviours dependent on the perception of space (Goodale & Milner, 1992).

### 4.3 Visual motion network connectivity

Visual area V5 represents the main cortical region of the visual motion network, and it specifically responds to motion stimuli (Braddick et al., 2001; Watson et al., 1993; Zeki et al., 1991). In the visually active state, the entire network showed hyper‐integration in VSS patients, both within its sub‐compartments—in the form of increased coupling from V5 to the striate and extrastriate visual cortices—as well as with other brain areas, mostly pertaining to the dorsal attentional network, such as the SPL and FEF. This network enables a top‐down selection of stimuli based on endogenous expectations and external cues (Corbetta, Kincade, Ollinger, McAvoy, & Shulman, 2000; Hopfinger, Buonocore, & Mangun, 2000), to best orient and allocate (Corbetta, Patel, & Shulman, 2008). It is functionally and anatomically distinct from the ventral attention network (VAN) (Corbetta & Shulman, 2002) which is responsible for refocusing attention to unattended and unexpected external stimuli (Fox, Corbetta, Snyder, Vincent, & Raichle, 2006).

It is interesting to find that several parts of the VAN, in particular the TPJ, angular gyrus and SMG (see Table 2), are conversely *less* integrated with the visual motion network in VSS patients in the active state. This could mean that in visual snow, the brain is exhibiting a reduced capacity to refocus visual attention to environmental stimuli, while it is allocating increased resources to the integration of internal and pre‐existing sensory information, via the DAN. It must be noted that our results of anticorrelation between V5 and the TPJ were limited to the right hemisphere; this is however not surprising considering the known lateralization of the VAN (Corbetta & Shulman, 2002).

### 4.4 Default mode and salience network dysfunction

The DMN represents a group of cortical areas that are specifically active during a non‐task state and suspended during goal directed behaviours (Raichle, 2010; Raichle et al., 2001). When placing seeds within the posterior nodes (PCC and precuneus) of the DMN, we found that patients with visual snow exhibited functional disruptions of its activity, both during the resting and stimulated states. This was represented by an increased connectivity at rest between the precuneus and FEFs (part of the DAN), and by a hyperintegration within the PCC itself in response to the visual stimulus. It was also confirmed by the anti‐correlations found between V5 and the PCC and between the PCC and the cerebellum, again in an area specifically associated with DAN (Guell, Schmahmann, Gabrieli, & Ghosh, 2018).

Further, our results found significant disruptions within the salience network (Dosenbach et al., 2006; Seeley et al., 2007), showing that the ACC and the anterior insula had abnormal coupling when the VSS brain was requested to process a normal external stimulus (Figure 3). In normal conditions, the anterior insula and the ACC function in unison to guide behaviour, by selecting the most relevant stimuli that reach the brain. The anterior insula in particular, is considered an integration hub for dynamic interactions between the other large‐scale brain networks (Menon & Uddin, 2010), constantly modulating a switch between the internally oriented self‐related cognition of the DMN and the externally directed attention of the DAN (Menon, 2011).

In this view, if we take together our findings of reduced connectivity between lower and higher hierarchical nodes of the visual pathway, of altered coupling between the DMN and DAN and of aberrant coupling within the salience network itself, it is thus possible to hypothesise a disruption within the normal integration of internal stimuli and of the processing of salient stimuli from the outside world, in VSS. This dysfunctional salience, facilitated by the hyper‐integration of the visual motion network and its reduced connections to the DMN and VAN, could perhaps cause the brain to misattribute salience to internal stimuli that would normally be considered as irrelevant, and to not appropriately ‘switch’ between internal and external attention, thus causing a constant, moving, ‘noise‐like’ perception. Whether these disruptions are due to aberrant nodes or aberrant architecture within the brain networks, and whether they are in fact a *primum movens* for the genesis of visual snow perception or rather a down‐stream effect of the perception itself, will need to be determined by future studies.

### 4.5 Migraine and visual snow

Two‐thirds of the VSS patents had a concomitant history of migraine; of these, seven reported visual aura as well (Table 1). VSS is highly comorbid with migraine and has even been classified as one of its possible complications (Headache Classification Committee of the International Headache Society (IHS), 2018). In this study we decided against a strict selection of VSS patients without migraine, as we thought this sub‐cohort would not be representative of the disorder. Further, functional connectivity studies focused on the visual system of migraineurs have shown results that are opposite to our current findings. One study found decreased connectivity between the sensorimotor network and the visual cortex and increased connectivity between the DMN and the visual cortices (Amin et al., 2016). Another found reduced connectivity between the DMN and the visuo‐spatial system (Coppola et al., 2016). Further, the fact that none of the VSS patients were in the ictal migraine phase during scanning, and most importantly our post hoc analysis accounting for migraine presence showing that relevant findings remained significant, seem to suggest that the connectivity findings presented here are mostly related to VSS.

### 4.6 Limitations

The main analysis in the study did not specifically investigate all regions of interest bilaterally. This was done initially to avoid errors due to multiple testing. Given the strongly significant results, we ran a post hoc comparison of the main seeds of interest in the contralateral hemisphere, that has been detailed in the supplementary material. From this analysis, it appears that the main VSS‐driven connectivity changes generalise to both hemispheres, rather than being specific to one or the other, with the main important exception of the altered connectivity between V5 and the temporo‐parietal junction, which as part of the ventral attentional network is known to have a lateralized, mostly right‐sided function.

Another important limitation is due to the fact that, as specified in the previous paragraph, our study included VSS patients with comorbid migraine, both with and without photophobia. Given that this symptom is common to both conditions, it is possible that it might have somewhat confounded our results, making it difficult to disentangle fully the underlying migraine biology from that of VSS. Even though the post hoc analysis accounting for migraine presence partially addresses this, it would nonetheless have been useful to perform a formal comparison between patients with and without migraine, and with and without photophobia. We did not, however, feel this was possible in the present study due to an inherent lack of power, given that over 60% of the patient cohort reported photophobia and/or migraine. Further, seven patients also reported a history of visual aura, which could have represented another potential confounder in the analysis.

## 5 CONCLUSIONS

In conclusion, our results suggest that VSS is characterised by a complex disturbance in the interaction of multiple brain systems. This dysfunction particularly involves the pre‐cortical and cortical visual pathway, the visual motion network, the attentional networks and finally the salience network; further, it does not depend on the activity state of the brain. What we observed suggests that there is a disruption in the filtering and integration of incoming sensory visual stimuli, versus the modulation of internally generated visual information. Future studies will help determine if this fingerprint of altered network dysfunction, indeed represents the main pathophysiological mechanism underlying the symptoms of this complex and disabling condition. In particular, it will be important to distinguish the impact of concomitant migraine, photophobia and aura on these findings, especially with regard to changes in connectivity affecting the visual system.

Given that no widely applicable treatment seems to fully suppress the symptoms of VSS, achieving an improved understanding of its underlying neurobiology is important, as it aids clinicians in explaining the condition to affected patients and also directs future research to more targeted approaches. In particular, the finding that diffuse brain networks are implicated in the genesis of VSS, could have the clinical implication of redirecting treatment from generic pharmacological interventions to a more focused modulation of brain function, possibly through techniques such as neuromodulation or neurofeedback.

## CONFLICT OF INTERESTS

The authors declare no competing financial interests relevant for this manuscript.

## Supporting information


**Appendix** S1: Supporting information

## Data Availability

The data that support the findings of this study are available upon reasonable request from the corresponding author.
