# Supplementary material for: Disrupted connectivity within visual, attentional and salience networks in the visual snow syndrome
Source: Hum Brain Mapp. 2021 Jan 15;42(7):2032–44. doi: 10.1002/hbm.25343 (PMC8046036; doi:10.1002/hbm.25343)
Supplement: Supplementary file 1 — Appendix S1: Supporting information [file HBM-42-2032-s001.docx]

**Disrupted connectivity within visual, attentional and salience networks in the visual snow syndrome**

**Supplementary material**

1. Image showing the eight regions of interest, with centre of mass coordinates (x; y; z) and size (k) for each individual seed.

Anatomical ROIs: Pv = right pulvinar; V1 = right primary visual area V1; V5 = right motion area V5; LG = right lingual gyrus;

Functional ROIs: Cb = left cerebellum lobule VI; pMCC/PCC = posterior midcingulate cortex/posterior cingulate cortex; PCu = left precuneus; IN = right insula.


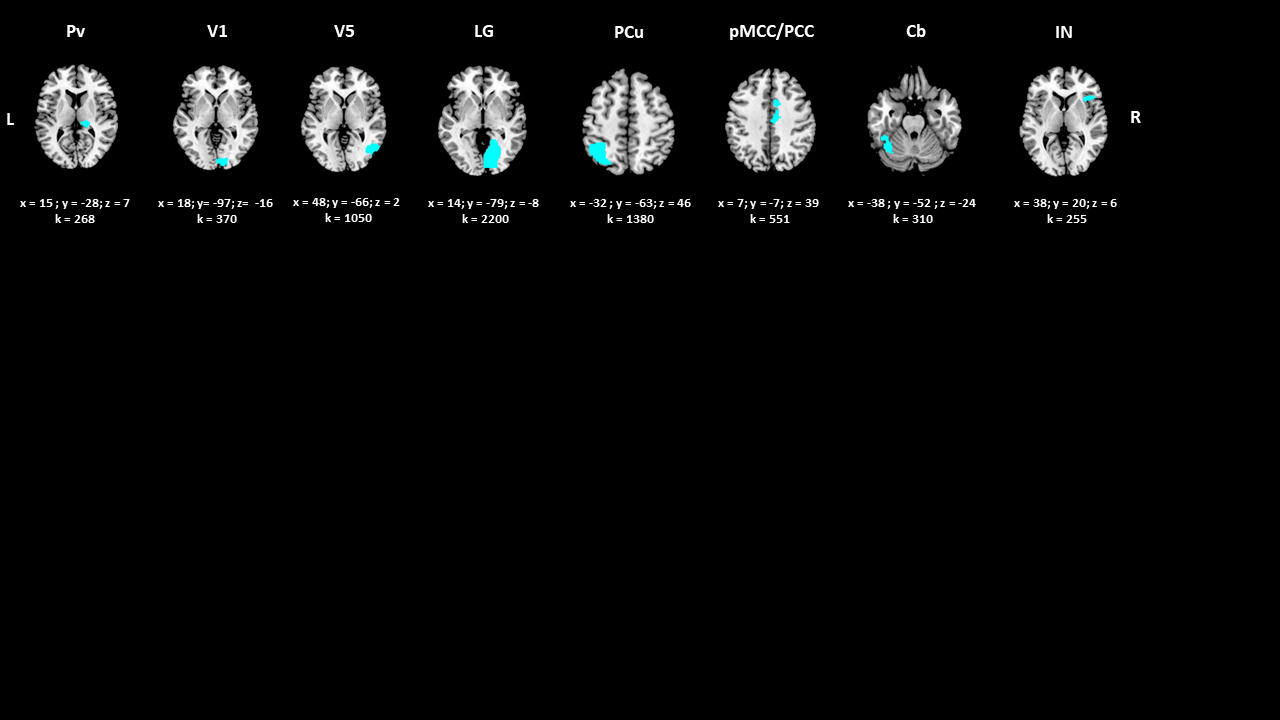


1. Detailed description results of *post-hoc* analysis investigating contralateral connectivity from the main anatomical regions of interest (Pv, V1, V5 and LG) and comparison to main results; a summary of this analysis can also be found in Table 3 of the main manuscript. The comparison was not run for functional ROIs (Cb, pMCC/PCC, PCu and IN), as these were already lateralized based on the results from a preliminary pseudo-continuous arterial spin labelling analysis.

Description of results:

- As for the right hemisphere, no significant changes in functional connectivity between groups were found from the left LG seed (not shown in table).
- When analysing connectivity from the left Pv seed at rest, we found the same areas of increased FC to the right SMG and postcentral gyrus in patients, with no change in side. The decreased FC to the left caudate nucleus was also found (with decreased cluster forming threshold of *P =* 0.005), however, this was now lateralized to the left side. No differences in FC were found during the task.
- From the left V1 region, the area of altered connectivity at rest to the SMG and postcentral gyrus was found in VSS patients to be on the contralateral hemisphere (right instead of left) respect to the main analysis, with decreased cluster forming threshold of *P =* 0.005. During the task, the increased connectivity in VSS to the SMG, postcentral and precentral gyri was still present, on the ipsilateral hemisphere respect to the main analysis.
- From the left V5 seed in the activated state, there was an increased connectivity in VSS to the same occipital and parietal areas as in the main analysis, particularly V1-V2-V3. This cluster was however mostly lateralized to the left side, it had a smaller extension and remained significant only after reducing the cluster forming threshold to *P =* 0.005. The anticorrelation between V5 and the PCC/bilateral precuneus cluster was confirmed as in the main analysis, as well as between V5 and the right TPJ.
